# Association of High Vitamin D Status with Low Circulating Thyroid-Stimulating Hormone Independent of Thyroid Hormone Levels in Middle-Aged and Elderly Males

**DOI:** 10.1155/2014/631819

**Published:** 2014-02-16

**Authors:** Qingqing Zhang, Zhixiao Wang, Min Sun, Mengdie Cao, Zhenxin Zhu, Qi Fu, Yuan Gao, Jia Mao, Yanyun Li, Yun Shi, Fan Yang, Shuai Zheng, Wei Tang, Yu Duan, Xiaoping Huang, Wei He, Tao Yang

**Affiliations:** ^1^Department of Endocrinology, The First Affiliated Hospital of Nanjing Medical University, Nanjing, Jiangsu 210029, China; ^2^Department of Endocrinology and Metabolism, The Affiliated Jiangyin Hospital of Southeast University Medical College, Jiangyin, Jiangsu 214400, China

## Abstract

*Background*. A recent study has reported that high circulating 25-hydroxyvitamin D [25(OH)D] is associated with low circulating thyroid-stimulating hormone (TSH) levels, but only in younger individuals. The goal of the present study was to explore the relationship between vitamin D status and circulating TSH levels with thyroid autoimmunity and thyroid hormone levels taken into consideration in a population-based health survey of middle-aged and elderly individuals. *Methods*. A total of 1,424 Chinese adults, aged 41–78 years, were enrolled in this cross-sectional study. Serum levels of 25(OH)D, TSH, thyroid hormones, and thyroid autoantibodies were measured. *Results*. The prevalence of vitamin D insufficiency was 94.29% in males and 97.22% in females, and the prevalence of vitamin D deficiency was 55.61% in males and 69.64% in females. Vitamin D status was not associated with positive thyroid autoantibodies after controlling for age, gender, body mass index, and smoking status. Higher 25(OH)D levels were associated with lower TSH levels after controlling for age, FT4 and FT3 levels, thyroid volume, the presence of thyroid nodule(s), and smoking status in males. *Conclusion*. High vitamin D status in middle-aged and elderly males was associated with low circulating TSH levels independent of thyroid hormone levels.

## 1. Introduction

Vitamin D is recognized to be an essential element for bone metabolism and skeletal health; however, its deficiency can cause rickets in children as well as an increased propensity for osteoporosis [1]. In addition, it may also affect extraskeletal health. Indeed, vitamin D deficiency has been identified as a risk factor for diabetes mellitus [2, 3], cancers [4], multiple sclerosis [5] and other autoimmune diseases [6, 7], atherosclerosis [8], and infectious diseases [9].

Few past studies have reported the impact of vitamin D deficiency on autoimmune thyroid disease and demonstrated inconclusive results [10, 11]. Besides affecting the thyroid gland through immune-mediated processes, vitamin D has been shown to influence rat thyroid follicular cells by directly inhibiting thyrotropin-stimulated iodide uptake in a dose-dependent manner [12]. Recently, a population-based study has reported that high vitamin D status in younger individuals is associated with low circulating thyroid-stimulating hormone (TSH) [13]. However, it remains unknown as to why no relationship between vitamin D status and serum TSH levels in middle-aged and elderly individuals was found in this study. Therefore, in the present study, we examined the relationship between vitamin D status and circulating TSH levels in middle-aged and elderly individuals with thyroid autoimmunity, while taking thyroid function into considerationin addition to the relationship between vitamin D insufficiency and thyroid autoimmunity, the presence of thyroid nodule(s) and thyroid volume in a cross-sectional study.

## 2. Materials and Methods

### 2.1. Study Subjects

From June to December 2011, we recruited a total of 9,982 Chinese living in Gulou, Nanjing. The study was approved by the ethics committee of the First Affiliated Hospital of Nanjing Medical University. All patients provided informed consent and completed a standardized questionnaire. Blood samples were collected and stored at −80°C.

The present study used a subsample collected from September to November 2011 to detect serum 25-hydroxyvitamin D [25(OH)D] levels, thyroid function, and thyroid autoantibodies. Subjects with a history of thyroid disease, without complete informations or taking medications that affected their thyroid function, such as oral contraceptives, oestrogen, glucocorticoids and iodine, were excluded.

Further exclusion criteria included overt hypothyroidism (TSH > 5.29 *μ*IU/mL; free thyroxine (FT4) < 8.5 pmol/L) and overt hyperthyroidism (TSH < 0.35 *μ*IU/mL; FT4 > 22.5 pmol/L). A total of 1,424 participants were included in our study.

### 2.2. Laboratory Measurements

Serum samples were collected from all 1,424 participants in the morning and all venous blood samples after an overnight fast. The serum samples were used to measure FT4, FT3, TSH, and 25(OH)D levels. Only 1,279 and 1,357 serum samples were used to measure thyroid peroxidase antibody (TPOAb) and thyroglobulin antibody (TgAb), respectively. Serum 25(OH)D levels were assessed using an enzyme immunoassay (IDS, UK). Serum TSH, TPOAb, and TgAb levels were measured using a chemiluminescent immunoassay (AutoBio Co., Ltd., Zhengzhou, China). Euthyroidism was defined as the absence of hypo- or hyperthyroidism. Serum TPOAb of >40 IU/mL and/or TgAb of >110 IU/mL were considered autoantibody positivity. High TPOAb and TgAb titres were defined as arbitrary values greater than 200 IU/mL and 550 IU/mL (four times greater than the normal values), respectively. We set vitamin D insufficiency and deficiency at levels below 75 nmol/L (30 ng/mL) and 50 nmol/L (20 ng/mL), respectively. The presence of thyroid nodule(s) and size of the thyroid gland were determined by thyroid ultrasonography. The thyroid volume was estimated by multiplication of its thickness, width, length, and a corrective factor (0.479) [14].

### 2.3. Statistical Analysis

Continuous variables are presented as means ± standard deviation for continuous normally distributed variables and median (interquartile range) for nonnormally distributed variables. Categorical variables are presented as percentage. The Kolmogorov-Smirnov method was used to test for normality. Differences between two groups for continuous and categorical variables were separately assessed using the Student's *t*-test and *χ* ^2^-test. The distribution of TSH levels deviated significantly from a Gaussian distribution (*P* < 0.000), while the logarithms of TSH levels were found to follow an approximate Gaussian distribution (*P* = 0.065). Linear regression analysis was used to examine the relationship between vitamin D status and logTSH values. Furthermore, bivariate correlation analysis was performed to study the correlation between vitamin D status and logTSH values. All calculations were performed using SPSS 13.0 for Windows (Chicago, IL, USA). A probability (*P*) value of <0.05 was considered statistically significant for all tests.

## 3. Results

### 3.1. Clinical and Laboratory Characteristics

The clinical and laboratory characteristics of the included 1,424 subjects (863 females and 561 males; age, 41–78 years) are shown in Table 1. On an average, females had higher serum TSH levels and TgAb titres, but lower serum 25(OH)D levels and proportion of smokers than males (3.03 (range, 1.89–4.60) versus 2.06 (1.36–3.30) IU/mL, *P* < 0.01; 13.19 (7.90–35.66) versus 10.08 (6.87–19.22) IU/mL, *P* < 0.01; 42.90 (35.15–53.11) versus 47.50 (38.00–59.00) IU/mL, *P* < 0.01 and 1.85% versus 44.92%, *P* < 0.01, resp.). There were no significant differences in other laboratory characteristics between males and females. The overall median (interquartile range) serum 25(OH)D level was 44.68 (range, 36.2–55.3) nmol/L. The prevalence of vitamin D insufficiency was 94.29% in males and 97.22% in females, whereas the prevalence of vitamin D deficiency was 55.61% in males and 69.64% in females. In the present study, 138 (10.17%) subjects were positive for serum TgAb, of which 42 (30.43%) had high TgAb titres. A total of 152 (11.88%) subjects were positive for serum TPOAb, of which 55 (36.18%) had high TPOAb titres. The prevalence of thyroid nodule(s) was 36.03%.

### 3.2. Comparison of Clinical and Laboratory Characteristics between Vitamin D Insufficiency and Sufficiency Groups

Based on a cutoff value of 75 nmol/L (30 ng/mL), the subjects were grouped into vitamin D insufficiency and vitamin D sufficiency. Differences between the clinical and laboratory characteristics between the two groups are shown in Table 2. There were significantly fewer males in the vitamin D insufficiency group (38.67% versus 57.14%, *P* = 0.006) than in the vitamin D sufficiency group. The serum TSH level and TPOAb and TgAb titres in the vitamin D insufficiency group were significantly higher than those in the vitamin D sufficiency group. However, there were no significant differences in serum FT3 and FT4 levels, the presence of thyroid nodule(s), thyroid volume, and smoking status between the two groups. Multivariate logistic regression analysis revealed that vitamin D status was not associated with thyroid autoantibody positivity after controlling for age, gender, body mass index (BMI), and smoking status (Table 3). Based on a cutoff value of 50 nmol/L (20 ng/mL), the subjects were grouped into vitamin D deficiency and nonvitamin D deficiency. High TgAb titres, High TPOAb titres, and high TgAb and/or TPOAb titres were significantly more common in the vitamin D deficiency group compared with those in the nonvitamin D deficiency group (4.04% versus 1.43%, *P* = 0.008; 5.48% versus 2.18%, *P* = 0.005; 7.70% versus 3.60%, *P* = 0.004, resp.). And vitamin D deficiency was independently associated with high thyroid autoantibody titres (see Supplementary Table 1 in the Supplementary Material available online at http://dx.doi.org/10.1155/2014/631819).

### 3.3. Relationship between Vitamin D Status and Serum TSH Levels according to Gender

With regard to the relationship between vitamin D status and serum TSH level, it was found that higher 25(OH)D levels were associated with lower TSH levels independent of age, FT3 and FT4 levels, TPOAb and TgAb titres, thyroid volume, the presence of thyroid nodule(s), and smoking status in males (Beta = −0.166, *P* = 0.004; Table 4). In addition, the association was found between thyroid volume and serum TSH levels in males (Beta = −0.152, *P* = 0.009) as well as the presence of thyroid nodule(s) and serum TSH levels in females (Beta = −0.116, *P* = 0.023). However, there were no significant relationship between serum TSH, FT3 and FT4 levels, TgAb and TPOAb titres, and smoking status between genders.

### 3.4. Relationship between Vitamin D Status and Serum TSH Levels on the Basis of Negative Serum Thyroid Autoantibody Titres in Males

To determine the probable interaction between vitamin D status and thyroid autoantibody positivity on serum TSH levels, further analyses were performed based on positive serum TPOAb, TgAb and TPOAb, and/or TgAb titres. The concentration of 25(OH)D was negatively correlated with serum TSH levels only in subjects with negative serum TPOAb, TgAb and TPOAb and/or TgAb titres (*P* = 0.002, 0.005 and 0.002, resp.; Supplementary Table 2). Linear regression analysis showed that higher 25(OH)D levels were associated with lower TSH levels independent of age, serum thyroid hormone levels, thyroid volume, the presence of thyroid nodule(s), and smoking status in males with negative serum TPOAb, TgAb, and TPOAb and/or TgAb titres (*P* = 0.004, 0.004, and 0.002, resp.; Table 5), but not in females or subjects positive for serum thyroid autoantibodies (data not shown).

## 4. Discussion

In the present study, we explored the probable interaction between vitamin D status and thyroid autoantibodies on serum TSH levels in middle-aged and elderly Chinese population with euthyroidism. This report is the first, to the best of our knowledge, to show that a higher circulating 25(OH)D level was associated with lower TSH levels only in males with negative serum thyroid autoantibody titres independent of thyroid hormone levels.

The result that high vitamin D status was associated with low circulating TSH levels remained unknown. We hypothesized that vitamin D may influence the thyrotrophs by acting on vitamin D receptors, which are widely distributed through distinct portions of the brain system [15]. A previous study has showed that vitamin D modulated pituitary thyrotropin TSH secretion by binding to specific binding sites [16]. Smith et al. [17] also found that exogenous vitamin D administration significantly suppressed pituitary thyrotropin TSH secretion in the basal state. This study also found that serum TSH levels of middle-aged and elderly women were higher than those of same-age men, and this result was consistent with those of previous reports [18, 19]. This result may indicate that TSH secretion is regulated by sex hormones, genetic susceptibility, or environmental factors, which may also mediate the relationship between vitamin D status and serum TSH levels. Another previous study [20] has showed that circulating oestrogen could induce acute serum TSH suppression in males by acting on the pituitary gland, and vitamin D was shown to be an important factor in oestrogen biosynthesis of both female and male gonads [21]. However, oestrogen administration has been reported to both increase [22] and decrease [23] or have no effect on thyroid activity in females [24]. Therefore, it may be safely inferred that oestrogens have a complex relationship with serum TSH secretion in females, which may explain the cause of this relationship only in men. We did not find the relationship between vitamin D status and serum TSH levels in subjects with positive thyroid antibody titres, which may be due to the limited number of participants, especially when divided into two groups according to gender. Further clinical investigation with a larger sample is required to elucidate the effects of vitamin D on the thyrotrophs to provide further insight into this relationship.

Chailurkit et al. [13] reported that an increase in serum 25(OH)D concentration was independently associated with lower TSH, but only in younger (age, 15–44 years) individuals. However, we found that higher 25(OH)D levels were independently associated with lower TSH in males with euthyroidism aged over 40 years. The results of the present study and those of Chailurkit et al.'s study differed for some reasons. First, subjects with overt hyperthyroidism and hypothyroidism by measuring serum FT3, FT4 and TSH levels were excluded in our study, and the results of our study reflect the general population setting with euthyroidism. Second, Chailurkit et al. did not separately analyse the relationship between 25(OH)D and serum TSH levels in males and females. Third, thyroid volume, the presence of thyroid nodule(s), and smoking status were considered confounding factors in our statistical analyses. Lastly, ethnicity was previously reported to be related with TSH levels [18], which plausibly explained the difference between the present and Chailurkit's studies.

It is generally agreed that serum 25(OH)D levels of <75 nmol/L (30 ng/mL) should be considered as representative of vitamin D insufficiency, whereas serum 25(OH)D levels of <50 nmol/L (20 ng/mL) as an indicative of vitamin D deficiency [25]. Using these definitions, our data showed that the prevalence of vitamin D insufficiency was 94.3% in males and 97.3% in females, and the prevalence of vitamin D deficiency was 55.6% in males and 69.6% in females. However, there were no conclusive results on the impact of vitamin D deficiency on autoimmune thyroid disease [18, 26]. In addition, our study did not find a link between vitamin D status and thyroid autoantibody positivity after controlling for age, gender, BMI, and smoking status. However, vitamin D deficiency was independently associated with high-titre thyroid autoantibody positivity. We thought that this high-titre thyroid autoantibody positivity should be paid more attention.

There were a number of limitations to the present study. First, TSH receptor-stimulating antibodies were not measured. Second, our study focused on middle-aged and elderly individuals; therefore, the relationship between vitamin D status and serum TSH levels in younger individuals with negative serum thyroid autoantibodies remained unknown. Finally, because of the cross-sectional nature of the present study, the causative effect of vitamin D on serum TSH could not be readily determined.

## 5. Conclusion

There was a high prevalence of vitamin D insufficiency among healthy adults. This population-based study was the first, to the best of our knowledge, to report an association between vitamin D and serum TSH levels independent of thyroid hormone levels in middle-aged and elderly males with negative thyroid autoimmunity. Here, we demonstrated a link between vitamin D insufficiency and serum thyroid autoantibody levels; however, vitamin D status was not associated with positive thyroid autoantibody titres after controlling for age, gender, and smoking status. Therefore, further longitudinal studies are required to clarify the relationship between vitamin D and serum TSH levels, particularly in subjects with negative-serum thyroid autoantibody titres.

## Supplementary Material

Multivariate logistic regression analysis revealed that vitamin D status was associated with strong positive thyroid antibodies after controlling for age, gender, BMI, and smoking status.

## Figures and Tables

**Table 1:** Clinical and laboratory characteristics of the participants.

Characteristics	Male (*n* = 561)	Female (*n* = 863)	Total (*n* = 1424)
Age (years)	59.22 ± 9.03	59.04 ± 8.20	59.11 ± 8.53
BMI (kg/m^2^)	24.93 ± 3.38	24.66 ± 3.36	24.76 ± 3.37
Serum 25 (OH)D (nmol/L)	47.50 (38.00–59.00)	42.90 (35.15–53.11)*	44.68 (36.20–55.30)
Serum FT3 (pmol/L)	4.34 (3.99–4.69)	4.31 (3.98–4.72)	4.32 (3.98–4.69)
Serum FT4 (pmol/L)	15.87 (13.10–18.03)	15.81 (13.22–17.95)	15.83 (13.18–17.97)
Serum TSH (*μ*IU/mL)	2.06 (1.36–3.30)	3.03 (1.89–4.60)*	2.65 (1.62–4.10)
Serum TPOAb (IU/mL)	3.09 (1.07–11.08)	4.18 (1.24–16.28)	3.66 (1.15–14.34)
Serum TgAb (IU/mL)	10.08 (6.87–19.22)	13.19 (7.90–35.66)*	12.06 (7.31–27.00)
Thyroid volume (mL)	8.44 (6.82–10.06)	7.24 (5.83–8.82)	7.74 (6.16–9.40)
Presence of thyroid nodule(s) (*n* (%))	174 (31.02%)	339 (39.28%)*	513 (36.03%)
Current smokers (*n* (%))	252 (44.92%)	16 (1.85%)*	268 (18.82%)

*Significantly different from males, *P* < 0.01.

**Table 2:** Comparison of clinical and laboratory characteristics based on vitamin D insufficiency.

	Vitamin D insufficiency (1368)	Nonvitamin D insufficiency (56)	*P* value
Age (years)	59.07 ± 8.55	60.07 ± 8.11	0.371
Male sex (*n* (%))	529 (38.67%)	32 (57.14%)	0.006
BMI (kg/m^2^)	24.79 ± 3.40	24.21 ± 2.50	0.106
Serum FT3 (pmol/L)	4.39 (3.97–4.69)	4.48 (4.18–4.75)	0.094
Serum FT4 (pmol/L)	15.51 (13.14–17.93)	16.29 (14.01–18.79)	0.092
Serum TSH (*μ*IU/mL)	3.42 (1.62–4.18)	2.47 (1.54–3.24)	0.000
Serum TgAb (IU/mL)	84.26 (7.26–27.77)	21.12 (8.38–22.40)	0.000
Serum TPOAb (IU/mL)	40.45 (1.17–14.33)	16.77 (0.99–14.99)	0.000
Thyroid volume (mL)	8.43 (6.12–9.35)	8.71 (6.53–10.62)	0.476
Presence of thyroid nodule(s) (*n* (%))	495 (36.18%)	18 (32.14%)	0.537
Current smokers (*n* (%))	814 (59.50%)	37 (66.07%)	0.326

**Table 3:** Determinants of positive serum thyroid antibodies.

	Serum TgAb		Serum TPOAb		Serum TgAb and/or TPOAb	
	Adjusted OR (95% CI)	*P* value	Adjusted OR (95% CI)	*P* value	Adjusted OR (95% CI)	*P* value
Age (years)	0.997 (0.974–1.022)	0.838	1.007 (0.983–1.032)	0.558	1.009 (0.987–1.030)	0.431
Male sex	2.738 (1.526–4.910)	0.001	2.200 (1.267–3.821)	0.005	2.524 (1.550–4.110)	0.000
BMI (kg/m^2^)	1.035 (0.974–1.099)	0.266	1.040 (0.979–1.106)	0.204	1.039 (0.984–1.097)	0.166
Smoking status	0.786 (0.368–1.676)	0.532	1.308 (0.685–2.499)	0.415	1.052 (0.578–1.916)	0.868
Serum 25(OH)D (nmol/L)	0.992 (0.979–1.004)	0.188	0.999 (0.987–1.010)	0.818	0.997 (0.986–1.009)	0.660

**Table 4:** Standardized regression coefficients of variables in relation to serum TSH according to gender.

	Male (561)		Female (863)	
	Beta	*P* value	Beta	*P* value
Age (years)	−0.012	0.836	−0.023	0.647
Serum FT3 (pmol/L)	0.069	0.244	−0.043	0.399
Serum FT4 (pmol/L)	−0.040	0.501	−0.066	0.201
Presence of TgAb (%)	0.029	0.636	0.098	0.060
Presence of TPOAb (%)	−0.002	0.971	0.056	0.284
Thyroid volume (mL)	−0.152	0.009	−0.056	0.262
Presence of thyroid nodule(s) (%)	−0.025	0.669	−0.116	0.023
Current smokers (*n* (%))	−0.054	0.361	−0.001	0.985
Serum 25(OH)D (nmol/L)	−0.166	0.004	−0.001	0.983

**Table 5:** Standardized regression coefficients of variables in relation to serum TSH in males with negative serum antibodies.

	Serum TgAb		Serum TPOAb		Serum TgAb and/or TPOAb	
	Beta	*P* value	Beta	*P* value	Beta	*P* value
Age (years)	0.024	0.686	0.019	0.749	0.006	0.921
Serum FT3 (pmol/L)	0.030	0.593	0.092	0.122	0.074	0.228
Serum FT4 (pmol/L)	0.020	0.726	−0.037	0.535	−0.028	0.648
Thyroid volume (mL)	−0.165	0.003	−0.170	0.004	−0.154	0.011
Presence of thyroid nodule(s) (%)	−0.035	0.532	−0.020	0.736	−0.024	0.696
Current smokers (*n* (%))	−0.064	0.264	−0.060	0.313	−0.067	0.277
Serum 25(OH)D (nmol/L)	−0.157	0.004	−0.166	0.004	−0.184	0.002
