# Supplementary material for: Association of High Vitamin D Status with Low Circulating Thyroid-Stimulating Hormone Independent of Thyroid Hormone Levels in Middle-Aged and Elderly Males
Source: Int J Endocrinol. 2014 Feb 16;2014:631819. doi: 10.1155/2014/631819 (PMC3947886; doi:10.1155/2014/631819)
Supplement: Supplementary file 1 — Multivariate logistic regression analysis revealed that vitamin D status was associated with strong positive thyroid antibodies after controlling for age, gender, BMI, and smoking status. [file 631819.f1.pdf]

SUPPLEMENTARY TABLE 1. DETERMINANTS OF STRONG POSITIVE THYROID ANTIBODIES

	Serum TgAb		Serum TPOAb		Serum TgAb and/or TPOAb	
	Adjusted OR (95% CI)	P -value	Adjusted OR (95% CI)	P -value	Adjusted OR (95% CI)	P -value
Age (years)	0.997(0.958-1.039)	0.901	0.964(0.928-1.002)	0.065	0.983(0.952-1.016)	0.310
Male sex	1.622(0.650-4.045)	0.300	1.310(0.566-3.028)	0.528	1.357(0.674-2.733)	0.392
BMI (kg/m^2)	1.052(0.954-1.161)	0.307	1.016(0.922-1.120)	0.745	1.051(0.969-1.139)	0.230
Smoking status	0.612(0.176-2.129)	0.440	0.635(0.218-1.855)	0.407	0.721(0.296-1.755)	0.471
Presence of vitamin D deficiency	2.801(1.141-6.878)	0.025	2.833(1.231-6.523)	0.014	2.168(1.144-4.108)	0.018

Supplementary Table 2. The Relationship Between Vitamin D and TSH Based on the Positivity of Serum Antibodies

	Serum TgAb		Serum TPOAb		Serum TgAb and/or TPOAb	
	Positive (138)	Negative (1219)	Positive (152)	Negative (1127)	Positive (213)	Negative (1010)
Correlation Coefficient	-0.102	-0.080	-0.096	-0.092	-0.070	-0.096
P-value	0.234	0.005	0.240	0.002	0.309	0.002
